# Caspase-8: Arbitrating Life and Death in the Innate Immune System

**DOI:** 10.3390/cells14040240

**Published:** 2025-02-07

**Authors:** Sahil Gupta, Monica Aida Lopez, Amin M. Ektesabi, James N. Tsoporis, Chirag M. Vaswani, Shil Y. Gandhi, Gregory D. Fairn, Claudia C. Dos Santos, John C. Marshall

**Affiliations:** 1 Faculty of Medicine, School of Medicine, The University of Queensland, Brisbane, QLD 4006, Australia; sahil.gupta@student.uq.edu.au (S.G.); s4710836@uq.edu.au (S.Y.G.); 2 Keenan Research Centre for Biomedical Science, St. Michael’s Hospital, Unity Health Toronto, Toronto, ON M5B 1W8, Canada; 19mal5@queensu.ca (M.A.L.); amin.ektesabi@mail.utoronto.ca (A.M.E.); jimtsoporis@sympatico.ca (J.N.T.); c.vaswani@mail.utoronto.ca (C.M.V.); gfairn@dal.ca (G.D.F.); claudia.dossantos@unityhealth.to (C.C.D.S.); 3 Institute of Medical Sciences, Temerty Faculty of Medicine, University of Toronto, Toronto, ON M5S 3K3, Canada; 4 Department of Physiology, Temerty Faculty of Medicine, University of Toronto, Toronto, ON M5S 3K3, Canada; 5 Department of Pathology, Dalhousie University, Halifax, NS B3H 4R2, Canada; 6 Department of Critical Care Medicine, St. Michael’s Hospital, Unity Health Toronto, Toronto, ON M5B 1W8, Canada

**Keywords:** caspase-8, apoptosis, inflammation, alternative splicing, post-translational modification

## Abstract

The canonical function of caspase-8 is to control timely cellular apoptosis to maintain tissue homeostasis and clear dysfunctional cells; however, emerging findings reveal novel, non-canonical roles of caspase in addition to regulating cellular apoptosis, including inflammatory response regulation, immune function, and cell differentiation. Furthermore, the functional versatility of caspase-8 is reported to be contingent on the presence and dimerization of various isoforms, which are produced through alternative splicing, altering its function and protein–protein interactions. Equally important are post-translational modifications, including phosphorylation and ubiquitination, which can act as a nexus to control caspase-8 activity and cellular localization. Here, we review the alternative splicing and post-translational modifications made to caspase-8 and discuss their influence on its canonical and non-canonical roles.

## 1. Introduction

Resilience in living organisms is a function of both inherent stability and the ability to adapt. Adaptability describes the capacity to change quickly in response to changing external circumstances, and so to maximize the chances of passing on a complex genetic program to future generations of life. Over the course of generations, this process is known as evolution and subject to the play of both external factors and random chance; however, over the lifespan of the individual organism, the processes are biologically explicit–encoded in the genome and shaped and conserved through eons of genetic history.

At the molecular level, evolutionary adaptability depends on the ability of genes to mutate, changing their expression and the structure and function of the proteins they encode, and the capacity of the protein products of those genes to undergo structural changes through differential splicing or post-translational modifications. At the cellular level, individual adaptability arises through the ability of cells to proliferate or, alternatively, to die through a variety of conserved processes. The ever-expanding repertoire of mechanisms of cell death speaks to the critical role that regulated cellular demise plays in homeostasis [1].

Apoptosis was the first described model of programmed cell death and was shown to be affected through the sequential activation of a family of enzymes termed *caspases*. Caspases have now been implicated in other forms of cell death, including necroptosis, immunogenic cell death, and pyroptosis [2,3]. They have also been shown to occupy pivotal roles in cellular activation and inflammatory function and so serve not only as cell death executioners but also as arbiters of cellular fate in response to acute biological threats. Caspase-8, originally identified as the apical enzyme in an extrinsic pathway of programmed cell death, exemplifies this biological duality in the fight or flight response of the cell [4,5,6,7,8].

## 2. The Caspase Family

Caspases are the mammalian homologs of the *Caenorhabditis elegans* death genes, Ced-3, Ced-4, and Ced-9 [9,10,11,12]. They function as cysteine–aspartic proteases that cleave their protein targets after aspartate residues in target proteins [1,13,14]. Cysteine residues found within caspase active sites serve as nucleophiles to hydrolyze peptide bonds—most commonly after the C-terminal linkage of aspartic acid, sometimes after glutamate, and rarely after phosphoserine residues [15,16,17].

**Figure 1:**
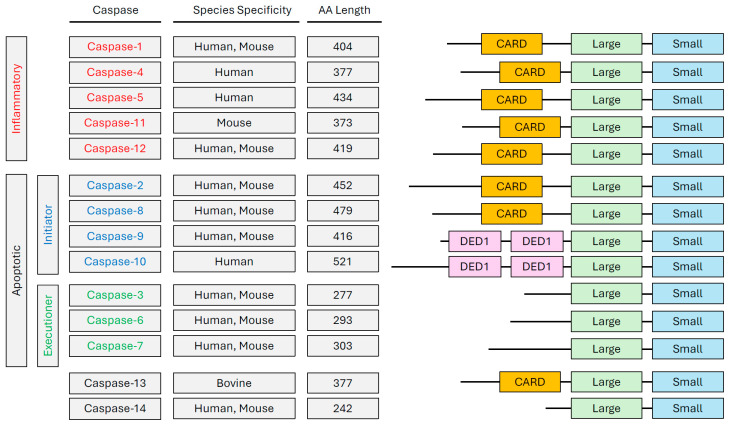
Categorized sequences of mammalian caspase proteases. There are 12 caspase proteins that are broadly divided into inflammatory (Caspase-1, -4, -5, -11, -12), initiator (Caspase-2, -8, -9, -10), and executioner (Caspase-3, -6, -7) groups. All inflammatory caspases, as well as caspase-2, -9, and -13, contain a CARD domain with a large and small subunit. Initiator caspase-8 and -10 both contain two death effector domains (DED1 and DED2) as well as a large and small subunit. Finally, executioner caspases-3, -6, and -7 only have a large and small subunit. Across all 14 mammalian caspase proteins, the large and small subunits collectively represent the catalytic domain with a small linker region between them. Caspase-13 and 14 are not categorized as either apoptotic or inflammatory caspases.

Fourteen different caspases have been reported in mammals thus far [18]. They have been divided into two clusters based on sequence similarity and function (Figure 1). The inflammatory caspase group comprises caspase-1, -4, -5, -11, and -12, whereas the apoptotic group includes caspase-2, -3, -6, -7, -8, -9, and -10. Pro-apoptotic caspases are further subclassified into initiator caspases (caspase-2, -8, -9, and -10) or effector/executioner caspases (caspase-3, -6, and -7) [19]. There is inter-species variability within the caspase family. Mice lack caspase-5 and caspase-10 and express an orthologue of caspase-4 as caspase-11. Caspase-13 exists exclusively in bovine species and is an orthologue of caspase-4 [20,21]. Caspase-14 is present only in keratinocytes and regulates differentiation, cornification, and skin protection; it is not categorized as either inflammatory or apoptotic [22].

Caspase-8 occupies a distinctive role within this intricate family. Its canonical role is as an initiator caspase, linking the engagement of a cell death receptor to the activation of apoptosis within the cell; however, it also exerts multiple non-apoptotic and pro-inflammatory effects arising from both its enzymatic activity and through post-translational modifications. Moreover, caspase-8 is expressed as multiple different isoforms that further impact its biological activity. We review these divergent biologic roles of caspase-8 and the role that post-translational modifications and differential splicing play in altering cellular function.

## 3. Caspase-8: Protein Structure and Biologic Activity

**Figure 2:**
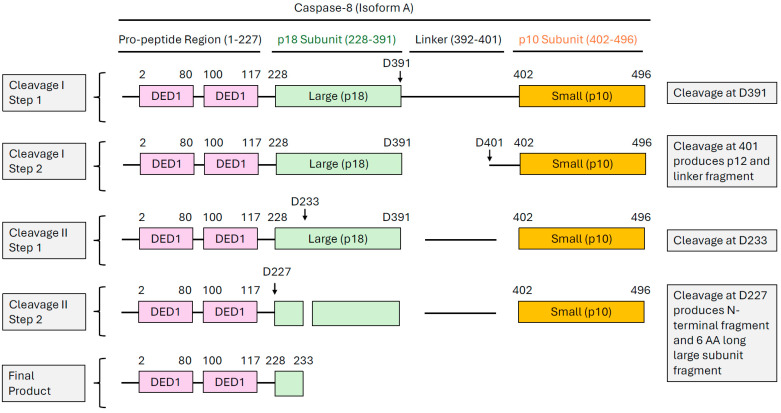
Sequential processing of caspase-8. This schematic represents the structural composition of the caspase-8A isoform. It contains two death effector domains (DED1 and DED2), one large p18 subunit, and one small p12 subunit. The numbers above the boxed regions reflect amino acid positions. There are two cleavage events that process caspase-8 into its catalytically active form. The first cleavage event occurs at D391, followed by subsequent cleavage at D401. This collectively produces an N-terminal fragment composed of two DEDs, a p18 subunit, and a small p12 fragment with a truncated linker region. The second cleavage event occurs at D233, which is followed by cleavage of D227. The final products produced from both cleavage events are an N-terminal fragment composed of two DEDs and a small six AA fragment of the large subunit.

The caspase-8 gene is located on band 2q33-34 of chromosome 2 and contains at least 11 exons over the course of 30 kilobase pairs in humans [23]. After translation, single-chain caspase-8 can localize to the cytoplasm [24], lamella of migrating cells [25], endosomal compartments [26], and microtubules and centrosomes [27]. Under normal physiological conditions, caspase-8 exists as an inactive dimeric zymogen composed of two death effector domains (DEDs) in the N-terminus: one large p18 protease subunit and one small p10 protease subunit. The p18 and p10 subunits collectively represent the catalytic domain of caspase-8 and are separated by a smaller linker region (Figure 2).

## 4. Pro-Apoptotic Activities of Caspase-8: The Death-Inducing Signaling Complex (DISC)

Caspase-8 was originally named FADD-like interleukin-β converting enzyme (FLICE) by virtue of its cysteine protease activity and its homology to the membrane-bound death receptor, Fas-associated death domain (FADD). It was identified as the apical enzyme of the extrinsic apoptotic pathway, activated by the binding of death ligands, such as Fas ligand (FasL) or tumor necrosis factor-alpha (TNF-α), to their respective death receptors on the cell surface. Binding triggers the assembly of a death-inducing signaling complex (DISC) consisting of the death receptor, adapter proteins, and pro-caspase-8. Formation of the DISC induces pro-caspase-8 molecules to undergo a conformational change, leading to activation through self-cleavage, releasing two catalytically active subunits–p 18 and p 10.

**Figure 3:**
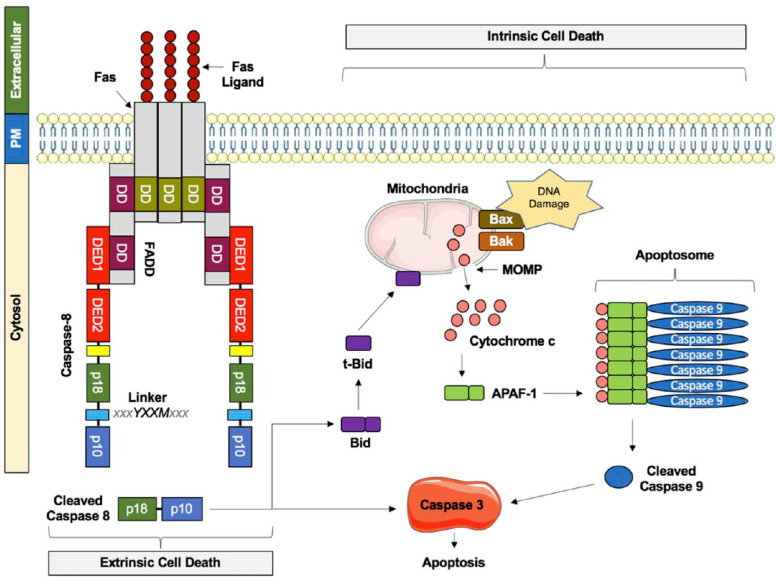
Extrinsic versus intrinsic cell death signaling. The extrinsic cell death pathway is triggered by the Fas ligand binding to the Fas receptor. This promotes Fas receptor trimerization and Fas-associated death domain (FADD) adaptor recruitment. FADD death domains (DD) further complex with caspase-8 death effector domains (DED), establishing the so-called death-induced signaling complex (DISC). Autocatalytic cleavage of caspase-8 activates p18 and p10 molecules to facilitate downstream caspase-3-dependent apoptosis. p18 and p10 fragments can also cleave bid, a BH3 containing Bcl-2 protein, to produce truncated bid (t-Bid). Translocation of t-Bid to the outer mitochondrial membrane promotes the leakage of cytochrome c into the cytoplasm through the mitochondrial outer membrane pore (MOMP) and initiates caspase-9-dependent intrinsic cell death signaling. The intrinsic cell death pathway is activated in response to stress signals such as DNA damage. Bak and Bax protein activation induces MOMP formation, releasing cytochrome c into the cytosolic compartment. Cytochrome c coupling to apoptosis protease activating factor-1 (APAF-1) and caspase-9 establishes the so-called apoptosome. Catalytic cleavage of caspase-9 results in caspase-3-dependent apoptotic signaling.

Heterodimeric p18:p10 caspase-8 directly cleaves and activates downstream effector caspases, including caspases-3, -6, and -7 [19]. These effector caspases, in turn, execute the final stages of apoptosis, degrading key cytostructural proteins, fragmenting DNA, and resulting in the formation of apoptotic bodies that are cleared by phagocytic cells. In addition to its role in the extrinsic pathway of apoptosis, caspase-8 can also trigger the intrinsic mitochondrial pathway by cleaving and activating cytoplasmic Bid. Once cleaved, truncated Bid (T-Bid) translocates to the mitochondria, where it induces the release of cytochrome c, thereby initiating the caspase-9-dependent intrinsic pathway of apoptosis [28] (Figure 3). The pro-apoptotic activity of pro-caspase-8 is regulated by its interaction with c-FLIP isoforms at the DISC. C-FLIP_L_ supports pro-caspase-8 activation and apoptosis, while c-FLIP_S_ inhibits it by forming heterodimers lacking enzymatic activity [29].

Caspase-8 exerts both pro-apoptotic and non-apoptotic activity within the cell. These divergent effects arise through both alternate splicing and post-translational modifications of the caspase-8 protein.

## 5. Regulation of Caspase-8 Function by Alternative Splicing

Alternative splicing is the process by which a single eukaryotic gene can produce multiple protein variants through selective inclusion or exclusion of specific exons during mRNA processing. The spliceosome, a complex of five small nuclear ribonucleoproteins (U1, U2, U4, U5, and U6 snRNPs) and auxiliary proteins, catalyzes the removal of non-coding introns and regulates the arrangement of exons to generate diverse mRNA transcripts [30]. Constitutive exons are flanked by highly conserved splice sites, ensuring their usual inclusion in the final mRNA sequence. Splicing factors recognize enhancing and silencing sequences within the pre-mRNA, determining the inclusion or exclusion of specific exons in the mature mRNA; however, point or frameshift mutations can disrupt splicing by altering conserved splice sites or regulatory sequences, leading to exon exclusion or aberrant splicing in the mature mRNA [31]. The consequence of differential splicing is the translation of a single gene into two or more functional protein variants.

Caspase-8 can be transcribed and translated into multiple splice variants. To date, ten caspase-8 isoforms (caspase-8 A-C, E-L, and s) have been reported [32,33,34]. Six of these variants (caspase-8A, 8B, 8C, 8F, 8G, and 8H) are composed of a homologous, 182 amino acid long N-terminal region that contains two DEDs. The C-terminus varies between these isoforms [32,35]. Caspase-8L was identified in human peripheral blood lymphocytes and represents the ninth caspase-8 isoform [36]. Through alternative splicing, 136 base pairs are inserted between exon 8 and exon 9 of the caspase-8 mRNA sequence, producing a premature stop codon. The insertion produces the caspase-8L variant which contains two DED domains but no catalytic domain, and so serves as a dominant negative isoform of caspase-8 that can inhibit the caspase cascade [36,37]. The latest caspase-8 isoform was identified in patients with acute leukemia and is called caspase-8s. This is the shortest caspase-8 variant, as it only expresses DED1 and a portion of DED2 [36].

**Figure 4:**
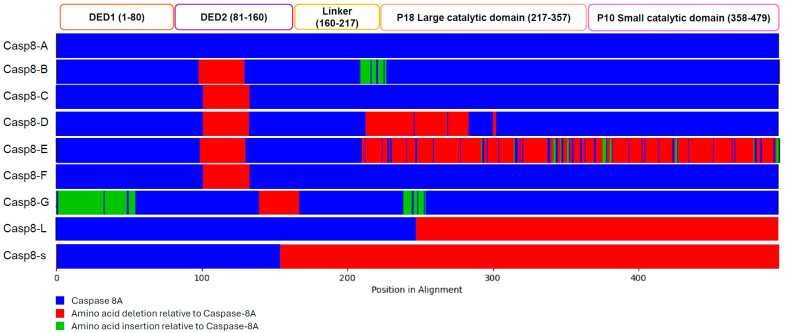
Sequence composition of caspase-8 isoforms. Regions that are inserted (green) or deleted (red) are marked relative to caspase-8A (A).

Although these 10 known variants are expressed in a variety of tissues, the primary variants caspase-8A and caspase-8B are mostly commonly detected at the protein level [32]. Sequence alignment of caspase-8A with B reveals a 32 amino acid insertion from position 102 to 134 and a 15 amino acid deletion from position 217 to 232 in caspase-8A relative to caspase-8B. This insertion disrupts a YXXM motif that is a potential SH2 binding site in the caspase-8A protein and results in a 17 amino acid difference between the two variants, equating to a 2 kDa molecular size difference such that caspase-8A has a molecular weight of 55 kDa, and caspase-8B a molecular weight of 53 kDa. The differences in these caspase-8 isoforms are summarized with reference to caspase-8A in Figure 4. Alternate splicing in exon 8 of caspase-8L results in the insertion of 136 base pairs [36]. This isoform mimics the structure of caspase-8A, conserving the DEDs but lacking the catalytic site of caspase-8. On the other hand, caspase-8s was found to have had a 106 bp deletion, which was responsible for a frameshift mutation containing a stop codon, creating a premature termination of the transcript [34]. Caspase-8A, caspase-8B, caspase-8C, caspase-8F, caspase-8G and caspase-8L undergo alternative splicing for exon 2 and exon 7. In contrast, caspase-8s and caspase-8E are shortened due to alternative splicing.

Alternative splicing of caspase-8 is cell-dependent and appears to be influenced by the activation state of the cell [38]. The activation of lymphocytes within patients experiencing systemic lupus erythematosus has been proposed to be responsible for a reduction in the expression of caspase-8L [33,36]. This contrasts with patients living with adult T-cell leukemia who have higher levels of caspase-8L in their peripheral blood mononuclear cells, which results in delayed apoptosis and increased cell inflammatory activity [39]. These reports could suggest that alternative splicing may be a regulatory mechanism to control levels of active caspase-8 in the cell [38].

## 6. Non-Canonical Roles of Caspase-8

In addition to its well-known role in apoptosis regulation, caspase-8 has been found to play diverse, non-canonical roles through interactions with intracellular signaling platforms or proteins. For example, caspase-8 can regulate the inflammatory and immune responses by promoting the production of pro-inflammatory cytokines, including interleukin-1β, through the activation of the NOD-, LRR- and pyrin domain-containing protein (NLRP3) inflammasome [40,41]. Caspase-8-dependent cleavage and activation of the NLRP3 trigger the assembly of the inflammasome complex to enable cytokine release. Conversely, caspase-8 activation can inhibit other signaling platforms, such as the necrosome, which is composed of receptor-interaction protein kinase 1 (RIPK1) and receptor-interacting protein kinase 3 (RIPK3). Caspase-8 cleaves RIPK1 at the Asp325 residue to destabilize the necrosome and prevent cell death [42]. In ex vivo studies, tyrosine phosphorylation of caspase-8 has been found to be pivotal for maintaining the integrity of epithelial barriers by regulating the kinetics of epithelial cell apoptosis. Jia et al. showed that caspase-8 is constitutively phosphorylated in resting epithelial cells [5]. Ex vivo co-culture of activated inflammatory neutrophils harvested from critically ill patients with secondary epithelial cell lines induced SHP-1-mediated caspase-8 dephosphorylation to hasten epithelial cell death [5]. These findings were further validated in secondary cell lines transfected with either phosphorylated or non-phosphorylatable caspase-8 mutant constructs [5]. Cells expressing non-phosphorylatable caspase-8 underwent accelerated apoptosis compared with cells transfected with phosphorylated caspase-8 [5].

Caspase-8 expression has also been reported to be vital for appropriate embryological development, cell differentiation, and homeostasis. In work by Kang et al.; conditional caspase-8 deficiency in murine endothelial cells degenerated yolk sac vasculature to elicit circulatory failure [43]. Conditional caspase-8 deletion in murine bone marrow cells further arrested hemopoietic progenitor cell function and ceased the differentiation of monocytes into macrophages [43]. Complimentary studies by Salmena et al. showed that caspase-8 expression is indispensable for maintaining T cell homeostasis. By analyzing lymphocyte surface marker expression on flow cytometry and anti-CD3 and anti-B220 histology staining, caspase-8 deficient mice were found to produce lower circulating, splenic, and lymph node T cells with a greater CD4^+^:CD8^+^ ratio compared with control mice [44]. Interestingly, while caspase-8 deficiency is embryonically lethal in mice, it is not in humans but can lead to severe clinical presentations, including immunodeficiency and very early-onset inflammatory bowel disease (IBD). A recent case report by Bazgir et al. described a two-year-old boy with caspase-8 deficiency presenting with a fever of unknown origin and dysentery refractory to antibiotic therapy, ultimately diagnosed as early-onset IBD [45]. Other case reports in two adolescents (11 and 12 years) [46] and two young adults (30 years) [47] also revealed caspase-8 deficiency manifests with autoimmune lymphoproliferative syndrome [46] and end-organ lymphocytic infiltration in the lungs, liver, spleen, bone marrow, and central nervous system [47]. Collectively, these reports show that caspase-8 plays an important role in human T-cell differentiation and response.

Caspase-8 is also known to regulate autophagy, a pro-survival cellular mechanism that recycles damaged organelles, protein aggregates, and pathogens within an autolysosome, promoting cell survival. Key proteins involved in autophagy, such as ATG7 and Beclin-1, are essential for this process [48]. Reduced expression of caspase-8 via RNA interference has been associated with features of autophagy, with the hypothesis that caspase-8 negatively modulates autophagy by interacting with autophagy-related proteins [49]. Caspase-8 inhibition has been shown to shift the cell death program towards autophagic cell death, a phenomenon not observed with the inhibition or suppression of caspases 1, 2, 3, 9, or 12 [49]; therefore, caspase-8 appears to play a distinctive role in preventing autophagic cell death, potentially through its regulation of the balance between apoptosis and autophagy.

## 7. Regulation of Caspase-8 Function by Post-Translational Modifications

Signal transduction in cells typically involves the alteration of protein–protein interactions through post-translational modifications (PTMs). Caspase-8 can undergo phosphorylation or ubiquitination to alter cell fate and function.

Caspase-8 catalytic activity in human neutrophils and cancer cell lines can be inhibited by phosphorylation [6,7]. Src-kinase-induced phosphorylation of caspase-8 at tyrosine 380 (Y380) inhibits caspase-8 release from the DISC to block caspase-3 cleavage [50,51]. In addition to Y380, Y448 serves as an alternative site for Src kinase binding to inhibit caspase-8 activation [4,8]. Conversely, binding of Src-homology domain 2 containing tyrosine phosphatase 1 (SHP-1) to caspase-8 can reverse Y380 and Y448 phosphorylation [7,52], releasing caspase-8 from the DISC to initiate caspase-3 dependent cell death signaling.

In contrast to Y380 and Y448 phosphorylation, phosphorylation of threonine 273 (T273) in the p18 domain of caspase-8 by polo-like 3 kinase (Plk-3) triggers cellular apoptosis [53]. Conversely, phosphorylation of T263 by ribosomal S6 kinase 2 (RSK2) induces caspase-8 ubiquitination, leading to downstream necroptosis [54]. In addition, phosphorylation of caspase-8 serine 364 (S364) by p38-mitogen-activated protein kinase (MAPK) inhibits caspase-8 activity to delay human neutrophil apoptosis and sustain an inflammatory state [55]. Collectively, T273, T263, and S364 are conserved evolutionary phosphorylation sites [53,54,55], suggesting that they serve key regulatory roles in cell death and possibly function. Similar consequences of caspase-8 phosphorylation have also been reported in cancer cell lines, primary breast tissue, and lymphocytes. Matthess et al. revealed that serine 387 (S387) phosphorylation by Cdk1/cyclin B1 blocks Asp374/Asp384 cleavage in the p10 subunit and so prolongs survival in mitotic cells by inhibiting caspase-8 dependent extrinsic apoptosis. These findings were replicated and validated using cyclin B1 siRNA, Cdk1 inhibitor (RO-3306), and non-phosphorylatable transfection (S387A) studies [56].

Caspase-8 can also undergo ubiquitination. Normally, the addition of 48 lysine residues [57] and rarely 63 lysine residues [58] localize target proteins for degradation through the 26S proteasome, whereas the addition of 63 lysine residues traditionally controls signal transduction through receptor endocytosis, protein trafficking, and signal activation [56,59]. Caspase-8 can be tagged with K48, signaling for its degradation through the proteasome, or K63 by TRIM13, to modulate caspase-8 translocation to autophagosomes and fusion with the lysosome to induce HEK293 cell death [60]. Similarly, ubiquitination plays a key role in regulating the half-life of caspase-8 in primary human cells. In human neutrophils, heat-shock protein 90 protects caspase-8 from ubiquitin-mediated degradation, sustaining interactions between tyrosine-phosphorylated caspase-8 and phosphoinositide-3-kinase that initiate and prolong anti-apoptotic signaling [50].

Other studies by Jin et al. using secondary cell lines demonstrated that cullin3-based E3 ligase-dependent caspase-8 K63 ubiquitination at K461 enabled p62-dependent caspase-8 aggregation and cytosolic translocation to drive caspase-8 autoproteolysis and apoptosis [61]. Jin et al. further revealed that CUL3-dependent caspase-8 polyubiquitination can be reversed by deubiquitinase A20 binding to caspase-8 [57]. Collectively, these studies show that caspase-8 can be degraded while undergoing activation, providing a further mechanism to regulate caspase-8-dependent apoptosis. Complimenting these findings, Gonzalvez et al. revealed that tumor necrosis factor receptor-associated factor 2 (TRAF2) binds and ubiquitinates caspase-8 by adding K48 tags downstream of cullin-3 at K224, K229, and K231 as an E3 ligase in the DISC to induce proteasomal degradation of caspase-8 in a time and threshold dependent manner [62].

Similarly, Xu et al. used gastric cancer cells to show that TRAF2-mediated K48 polyubiquitination of caspase-8 leads to its proteasomal degradation, reducing caspase-8 pro-apoptotic activity. This process stabilizes the integrity of the DR5-Cbl-b/c-Cbl-TRAF2 complex, thereby inhibiting TNF-related apoptosis-inducing ligand (TRAIL)-dependent cell death [62]. Beyond this intricate and complex mechanism regulating caspase-8 polyubiquitination, Ivanova et al. further showed that caspase-8 polyubiquitination can be amplified when caspase-8 interacts with TP53INP2, which serves as a scaffold for caspase-8 interaction with ubiquitin ligase TRAF6 through a TRAF6-interacting motif (TIM) and ubiquitin-interacting motif and promote TRAIL-dependent apoptosis [63]. Finally, Alturki et al. identified a negative feedback loop that facilitates the degradation of caspase-8, RIPK1, and other necrosomal proteins to limit necroptosis and inflammatory cytokine production through Triad3a-mediated K48 ubiquitination. They reported that this process occurs during early necrosome activation and functions independently of traditional RIPK1 ubiquitin editing enzymes, such as A20 [64]. Finally, it is important to recognize that other E3 ubiquitin ligases, such as Cullin 7 (CUL7), can block caspase-8 degradation to enable cancer survival [65]. Using human breast- and cervical cancer cell lines, Kong et al. demonstrated that CUL7 adds non-degradative polyubiquitin chains to caspase-8 at K215 to promote anti-apoptotic caspase-8 activity within the DISC [65] (Supplementary Table S1).

## 8. Conclusions

**Figure 5:**
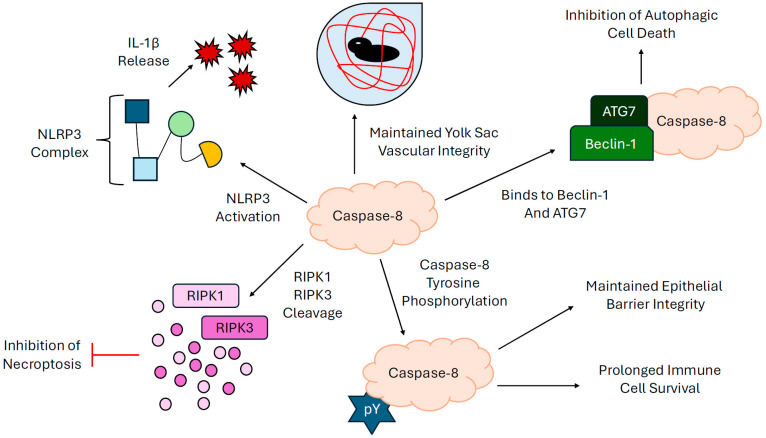
Cartoon summary of the non-canonical roles of caspase-8.

Caspase-8 is strategically located at the cell surface, where it is directly impacted by signals from the cellular microenvironment. A combination of the expression of multiple splice variants with differing effects on the progression of apoptosis and a variety of post-translational modifications that not only support or impede the progression of apoptosis but also support the activation of other intracellular signaling cascades supports a critical role for caspase-8 in regulating a dynamic cellular response to signals emanating from the extracellular environment (Figure 5). How this plasticity contributes to the pathology of disease and how these modifiable alterations might be manipulated to therapeutic benefit is emerging as an important frontier.

## Data Availability

No new data were created or analyzed in this study. Data sharing is not applicable to this article.

## Supplementary Materials

The following supporting information can be downloaded at: https://www.mdpi.com/article/10.3390/cells14040240/s1, Table S1: Summary table of post-translational modifications made to caspase-8 and the associated functional consequences.; Figure S1: Caspase-8 amino acid alignment relative to other caspase-8 isoforms.

## Abbreviations

DEDs	Death effector domains
DISC	Death-inducing signaling complex
FADD	Fas-associated death domain
FasL	Fas ligand
FLICE	FADD-like interleukin-β converting enzyme
FLIP	FLICE inhibitory protein
MAPKs	Mitogen-activated protein kinases
MLKL	Mixed lineage kinase domain-like
NF-κB	Nuclear factor kappa-light-chain-enhancer of activated B cells
NLRP3	NOD-, LRR- and pyrin domain-containing protein
Plk1	Polo-like kinase 1
PTMs	Post-translational modifications
RIPK1	Receptor-interacting protein kinase 1
SHP-1	Src-homology domain 2 containing tyrosine phosphatase 1
SUMO1	Small Ubiquitin-related Modifier-1
TNF-α	Tumor necrosis factor-alpha

## References

[1] Galluzzi L., Vitale I., Aaronson S.A., Abrams J.M., Adam D., Agostinis P., Alnemri E.S., Altucci L., Amelio I., Andrews D.W. (2018). Molecular mechanisms of cell death: Recommendations of the Nomenclature Committee on Cell Death 2018. Cell Death Differ..

[2] Shi J., Gao W., Shao F. (2017). Pyroptosis: Gasdermin-Mediated Programmed Necrotic Cell Death. Trends Biochem. Sci..

[3] Orozco S., Yatim N., Werner M.R., Tran H., Gunja S.Y., Tait S.W.G., Albert M.L., Green D.R., Oberst A. (2014). RIPK1 both positively and negatively regulates RIPK3 oligomerization and necroptosis. Cell Death Differ..

[4] Song H.J., Parodo J., Kapus A., Rotstein O.D., Marshall J.C. (2008). Dynamic regulation of neutrophil survival through tyrosine phosphorylation or dephosphorylation of caspase-8. J. Biol. Chem..

[5] Jia S.H., Parodo J., Charbonney E., Tsang J.L.Y., Jia S.Y., Rotstein O.D., Kapus A., Marshall J.C. (2014). Activated neutrophils induce epithelial cell apoptosis through oxidant-dependent tyrosine dephosphorylation of caspase-8. Am. J. Pathol..

[6] Gupta S., Lee C.M., Wang J.F., Parodo J., Jia S.H., Hu J., Marshall J.C. (2018). Heat-shock protein-90 prolongs septic neutrophil survival by protecting c-Src kinase and caspase-8 from proteasomal degradation. J. Leukoc. Biol..

[7] Cursi S., Rufini A., Stagni V., Condò I., Matafora V., Bachi A., Bonifazi A.P., Coppola L., Superti-Furga G., Testi R. (2006). Src kinase phosphorylates Caspase-8 on Tyr380: A novel mechanism of apoptosis suppression. EMBO J..

[8] Gupta S. (2020). Activation and Stabilization of the Caspase-8 Survivalsome Is Indispensable for Functional Toll-like Receptor 4 Dependent Signaling in Septic Neutrophils. Ph.D Thesis.

[9] Ellis H.M., Horvitz H.R. (1986). Genetic control of programmed cell death in the nematode C. elegans. Cell.

[10] Chinnaiyan A.M., O’Rourke K., Lane B.R., Dixit V.M. (1997). Interaction of CED-4 with CED-3 and CED-9: A molecular framework for cell death. Science.

[11] Hengartner M.O. (1996). Programmed cell death in invertebrates. Curr. Opin. Genet. Dev..

[12] Hengartner M.O., Horvitz H.R.C. (1994). elegans cell survival gene ced-9 encodes a functional homolog of the mammalian proto-oncogene bcl-2. Cell.

[13] Li J., Yuan J. (2008). Caspases in apoptosis and beyond. Oncogene.

[14] Julien O., Wells J.A. (2017). Caspases and their substrates. Cell Death Differ..

[15] Thornberry N.A., Rano T.A., Peterson E.P., Rasper D.M., Timkey T., Garcia-Calvo M., Houtzager V.M., Nordstrom P.A., Roy S., Vaillancourt J.P. (1997). A combinatorial approach defines specificities of members of the caspase family and granzyme B. Functional relationships established for key mediators of apoptosis. J. Biol. Chem..

[16] Stennicke H., Renatus M., Meldal M., Salvesen G. (2000). Internally quenched fluorescent peptide substrates disclose the subsite preferences of human caspases 1, 3, 6, 7 and 8—PubMed. Biochem. J..

[17] Seaman J.E., Julien O., Lee P.S., Rettenmaier T.J., Thomsen N.D., Wells J.A. (2016). Cacidases: Caspases can cleave after aspartate, glutamate and phosphoserine residues. Cell Death Differ..

[18] Zheng M., Karki R., Vogel P., Kanneganti T.D. (2020). Caspase-6 Is a Key Regulator of Innate Immunity, Inflammasome Activation, and Host Defense. Cell.

[19] Shalini S., Dorstyn L., Dawar S., Kumar S. (2014). Old, new and emerging functions of caspases. Cell Death Differ..

[20] Eckhart L., Ballaun C., Hermann M., VandeBerg J.L., Sipos W., Uthman A., Fischer H., Tschachler E. (2008). Identification of novel mammalian caspases reveals an important role of gene loss in shaping the human caspase repertoire. Mol. Biol. Evol..

[21] McIlwain D.R., Berger T., Mak T.W. (2013). Caspase Functions in Cell Death and Disease. Cold Spring Harb. Perspect. Biol..

[22] Denecker G., Hoste E., Gilbert B., Hochepied T., Ovaere P., Lippens S., Van den Broecke C., Van Damme P., D’Herde K., Hachem J.P. (2007). Caspase-14 protects against epidermal UVB photodamage and water loss. Nat. Cell Biol..

[23] Grenet J., Teitz T., Wei T., Valentine V., Kidd V.J. (1999). Structure and chromosome localization of the human CASP8 gene. Gene.

[24] Chang D.W., Xing Z., Capacio V.L., Peter M.E., Yang X. (2003). Interdimer processing mechanism of procaspase-8 activation. EMBO J..

[25] Barbero S., Barilà D., Mielgo A., Stagni V., Clair K., Stupack D. (2008). Identification of a Critical Tyrosine Residue in Caspase 8 That Promotes Cell Migration. J. Biol. Chem..

[26] Lee K.H., Feig C., Tchikov V., Schickel R., Hallas C., Schütze S., Peter M.E., Chan A.C. (2006). The role of receptor internalization in CD95 signaling. EMBO J..

[27] Mielgo A., Torres V.A., Clair K., Barbero S., Stupack D.G. (2009). Paclitaxel promotes a caspase 8-mediated apoptosis via death effector domain association with microtubules. Oncogene.

[28] Yuan J., Ofengeim D. (2023). A guide to cell death pathways. Nat. Rev. Mol. Cell Biol..

[29] Hughes M.A., Powley I.R., Jukes-Jones R., Horn S., Feoktistova M., Fairall L., Schwabe J.W.R., Leverkus M., Cain K., MacFarlane M. (2016). Co-operative and Hierarchical Binding of c-FLIP and Caspase-8: A Unified Model Defines How c-FLIP Isoforms Differentially Control Cell Fate. Mol. Cell.

[30] Paronetto M.P., Passacantilli I., Sette C. (2016). Alternative splicing and cell survival: From tissue homeostasis to disease. Cell Death Differ..

[31] Wang Y., Liu J., Huang B., Xu Y.-M., Li J., Huang L.-F., Lin J., Zhang J., Min Q.-H., Yang W.-M. (2015). Mechanism of alternative splicing and its regulation. Biomed. Rep..

[32] Scaffidi C., Medema J.P., Krammer P.H., Peter M.E. (1997). FLICE Is Predominantly Expressed as Two Functionally Active Isoforms, Caspase-8/a and Caspase-8/b. J. Biol. Chem..

[33] Horiuchi T., Himeji D., Tsukamoto H., Harashima S.I., Hashimura C., Hayashi K. (2000). Dominant expression of a novel splice variant of caspase-8 in human peripheral blood lymphocytes. Biochem. Biophys. Res. Commun..

[34] Xu Z., Tang K., Wang M., Rao Q., Liu B., Wang J. (2009). A New Caspase-8 Isoform Caspase-8s Increased Sensitivity to Apoptosis in Jurkat Cells. J. Biomed. Biotechnol..

[35] Boldin M.P., Goncharov T.M., Goltsev Y.V., Wallach D. (1996). Involvement of MACH, a novel MORT1/FADD-interacting protease, in Fas/APO-1- and TNF receptor-induced cell death. Cell.

[36] Himeji D., Horiuchi T., Tsukamoto H., Hayashi K., Watanabe T., Harada M. (2002). Characterization of caspase-8L: A novel isoform of caspase-8 that behaves as an inhibitor of the caspase cascade. Blood.

[37] Fulda S. (2009). Caspase-8 in cancer biology and therapy. Cancer Lett..

[38] Eckhart L., Henry M., Santos-Beneit A.M., Schmitz I., Krueger A., Fischer H., Bach J., Ban J., Kirchhoff S., Krammer P.H. (2001). Alternative splicing of caspase-8 mRNA during differentiation of human leukocytes. Biochem. Biophys. Res. Commun..

[39] Nakano K., Iwanaga M., Utsunomiya A., Uchimaru K., Watanabe T. (2019). Functional Analysis of Aberrantly Spliced Caspase8 Variants in Adult T-Cell Leukemia Cells. Mol. Cancer Res..

[40] Antonopoulos C., Russo H.M., El Sanadi C., Martin B.N., Li X., Kaiser W.J., Mocarski E.S., Dubyak G.R. (2015). Caspase-8 as an Effector and Regulator of NLRP3 Inflammasome Signaling. J. Biol. Chem..

[41] Gringhuis S.I., Kaptein T.M., Wevers B.A., Theelen B., Van Der Vlist M., Boekhout T., Geijtenbeek T.B.H. (2012). Dectin-1 is an extracellular pathogen sensor for the induction and processing of IL-1β via a noncanonical caspase-8 inflammasome. Nat. Immunol..

[42] Newton K., Wickliffe K.E., Dugger D.L., Maltzman A., Roose-Girma M., Dohse M., Kőműves L., Webster J.D., Dixit V.M. (2019). Cleavage of RIPK1 by caspase-8 is crucial for limiting apoptosis and necroptosis. Nature.

[43] Kang T.-B., Ben-Moshe T., Varfolomeev E.E., Pewzner-Jung Y., Yogev N., Jurewicz A., Waisman A., Brenner O., Haffner R., Gustafsson E. (2004). Caspase-8 serves both apoptotic and nonapoptotic roles. J. Immunol..

[44] Salmena L., Lemmers B., Hakem A., Matysiak-Zablocki E., Murakami K., Billie Au P.Y., Berry D.M., Tamblyn L., Shehabeldin A., Migon E. (2003). Essential role for caspase 8 in T-cell homeostasis and T-cell-mediated immunity. Genes. Dev..

[45] Bazgir N., Tahvildari A., Chavoshzade Z., Jamee M., Golchehre Z., Karimi A., Dara N., Fallahi M., Keramatipour M., Karamzade A. (2023). A rare immunological disease, caspase 8 deficiency: Case report and literature review. Allergy Asthma Clin. Immunol..

[46] Chun H.J., Zheng L., Ahmad M., Wang J., Speirs C.K., Siegel R.M., Dale J.K., Puck J., Davis J., Hall C.G. (2002). Pleiotropic defects in lymphocyte activation caused by caspase-8 mutations lead to human immunodeficiency. Nature.

[47] Niemela J., Kuehn H.S., Kelly C., Zhang M., Davies J., Melendez J., Dreiling J., Kleiner D., Calvo K., Oliveira J.B. (2015). Caspase-8 Deficiency Presenting as Late-Onset Multi-Organ Lymphocytic Infiltration with Granulomas in two Adult Siblings. J. Clin. Immunol..

[48] Tsapras P., Nezis I.P. (2017). Caspase involvement in autophagy. Cell Death Differ..

[49] Yu L., Alva A., Su H., Dutt P., Freundt E., Welsh S., Baehrecke E.H., Lenardo M.J. (2004). Regulation of an ATG7-beclin 1 program of autophagic cell death by caspase-8. Science.

[50] Powley I.R., Hughes M.A., Cain K., MaCfarlane M. (2016). Caspase-8 tyrosine-380 phosphorylation inhibits CD95 DISC function by preventing procaspase-8 maturation and cycling within the complex. Oncogene.

[51] Tsang J.L.Y., Jia S.H., Parodo J., Plant P., Lodyga M., Charbonney E., Szaszi K., Kapus A., Marshall J.C. (2016). Tyrosine Phosphorylation of Caspase-8 Abrogates Its Apoptotic Activity and Promotes Activation of c-Src. PLoS ONE.

[52] Kurokawa M., Kornbluth S. (2009). Caspases and Kinases in a Death Grip. Cell.

[53] Peng C., Cho Y.Y., Zhu F., Zhang J., Wen W., Xu Y. (2011). Phosphorylation of Caspase-8 (Thr-263) by Ribosomal S6 Kinase 2 (RSK2) Mediates Caspase-8 Ubiquitination and Stability. J. Biol. Chem..

[54] Alvarado-Kristensson M., Melander F., Leandersson K., Rönnstrand L., Wernstedt C., Andersson T. (2004). p38-MAPK Signals Survival by Phosphorylation of Caspase-8 and Caspase-3 in Human Neutrophils. J. Exp. Med..

[55] Matthess Y., Raab M., Knecht R., Becker S., Strebhardt K. (2014). Sequential Cdk1 and Plk1 phosphorylation of caspase-8 triggers apoptotic cell death during mitosis. Mol. Oncol..

[56] Kleiger G., Mayor T. (2014). Perilous journey: A tour of the ubiquitin-proteasome system. Trends Cell Biol..

[57] Jin Z., Li Y., Pitti R., Lawrence D., Pham V.C., Lill J.R., Ashkenazi A. (2009). Cullin3-based polyubiquitination and p62-dependent aggregation of caspase-8 mediate extrinsic apoptosis signaling. Cell.

[58] Saeki Y., Kudo T., Sone T., Kikuchi Y., Yokosawa H., Toh-e A., Tanaka K. (2009). Lysine 63-linked polyubiquitin chain may serve as a targeting signal for the 26S proteasome. EMBO J..

[59] Yang W.L., Zhang X., Lin H.K. (2010). Emerging role of Lys-63 ubiquitination in protein kinase and phosphatase activation and cancer development. Oncogene.

[60] Tomar D., Prajapati P., Sripada L., Singh K., Singh R., Singh A.K., Singh R. (2013). TRIM13 regulates caspase-8 ubiquitination, translocation to autophagosomes and activation during ER stress induced cell death. Biochim. Biophys. Acta (BBA) Mol. Cell Res..

[61] Dickens L.S., Boyd R.S., Jukes-Jones R., Hughes M.A., Robinson G.L., Fairall L., Schwabe J.W.R., Cain K., MacFarlane M. (2012). A death effector domain chain DISC model reveals a crucial role for caspase-8 chain assembly in mediating apoptotic cell death. Mol. Cell.

[62] Xu L., Zhang Y., Qu X., Che X., Guo T., Li C., Ma R., Fan Y., Ma Y., Hou K. (2017). DR5-Cbl-b/c-Cbl-TRAF2 complex inhibits TRAIL-induced apoptosis by promoting TRAF2-mediated polyubiquitination of caspase-8 in gastric cancer cells. Mol. Oncol..

[63] Ivanova S., Polajnar M., Narbona-Perez A.J., Hernandez-Alvarez M.I., Frager P., Slobodnyuk K., Plana N., Nebreda A.R., Palacin M., Gomis R.R. (2019). Regulation of death receptor signaling by the autophagy protein TP53INP2. EMBO J..

[64] Alturki N.A., McComb S., Ariana A., Rijal D., Korneluk R.G., Sun S.C., Alnemri E., Sad S. (2018). Triad3a induces the degradation of early necrosome to limit RipK1-dependent cytokine production and necroptosis. Cell Death Dis..

[65] Kong Y., Wang Z., Huang M., Zhou Z., Li Y., Miao H., Wan X., Huang J., Mao X., Chen C. (2019). CUL7 promotes cancer cell survival through promoting Caspase-8 ubiquitination. Int. J. Cancer.

