# Supplementary material for: Caspase-8: Arbitrating Life and Death in the Innate Immune System
Source: Cells. 2025 Feb 7;14(4):240. doi: 10.3390/cells14040240 (PMC11853578; doi:10.3390/cells14040240)
Supplement: Supplementary file 1 [file cells-14-00240-s001.zip › cells-3396714-supplementary.pdf]

Supplementary Table S1. Summary table of post-translational modifications made to caspase-8 and the associated functional consequences.

Post-Translational Modification	Mechanism	Consequence
Phosphorylation at Y380 & Y448	Src kinase phosphorylates caspase-8 at Y380 and Y448 to prevent release of caspase-8 from the DISC	Inhibits caspase-8 activation and downstream caspase-3 cleavage to block apoptosis
Dephosphorylation at Y380 & Y448	SHP-1 dephosphorylates caspase-8 at Y380 and Y448 to initiate caspase-3 dependent cell death	Enables caspase-8 release from the DISC to trigger apoptosis
Phosphorylation at T273	Polo-like kinase (Plk-3) phosphorylates caspase-8 at T283 in the p18 domain	Triggers apoptosis
Phosphorylation at T263	Ribosomal S6 kinase 2 (RSK2) phosphorylates caspase-8 at T263	Induces caspase-8 ubiquitination to promote necroptosis
Phosphorylation at S364	P38-MAPK phosphorylates caspase-8 at S364	Inhibits caspase-8 activity to delay neutrophil apoptosis and sustain an inflammatory state
Phosphorylation at S387	Cdk1/cyclin B1 phosphorylates caspase-8 at S387	Blocks caspase-8 cleavage at Asp374/Asp384 to prolong mitotic survival and inhibiting caspase-8 dependent extrinsic cell death
K48 Ubiquitination	TRAF2 and Cullin3-based E3 ligases add K48 polyubiquitin chains at K224, K229, K231	Induces caspase-8 for proteasomal degradation in a time and threshold dependent manner
K63 Ubiquitination	TRIM13 and Cullin-3 add K63 polyubiquitin chains at K461	Modulates caspase-8 cytosolic translocation and proteolysis to trigger cell death
Ubiquitination at K215	CUL7 adds non-degradative polyubiquitin chains to caspase-8 at K215	Enhances anti-apoptotic caspase-8 activity within the DISC to promote cancer survival
Negative Feedback Regulation	Triad3a-mediated K48 ubiquitination degrades caspase-8, RIPK1, and other necrosomal proteins	Limits necroptosis and inflammatory cytokine production

Casp8A vs Casp8A

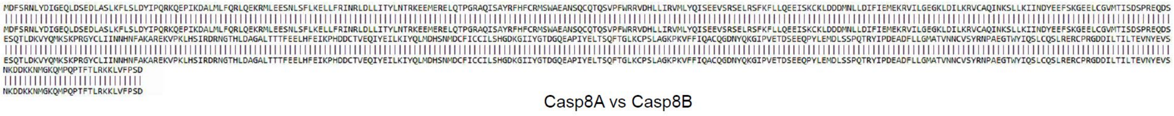

Casp8A vs Casp8B

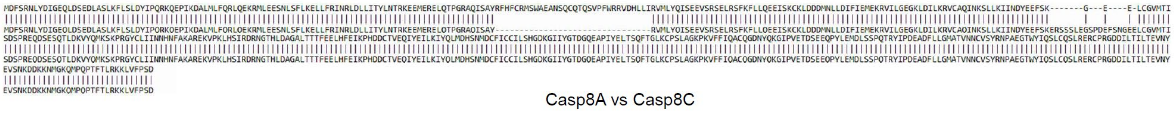

Casp8A vs Casp8C

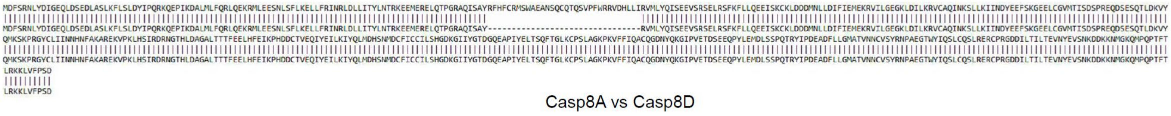

Casp8A vs Casp8D

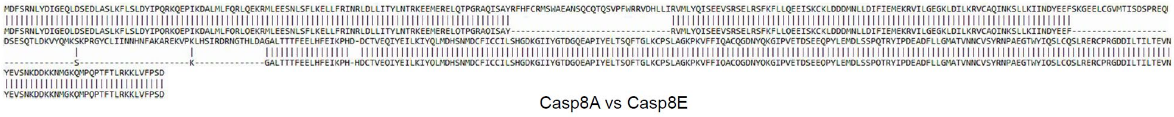

Casp8A vs Casp8E

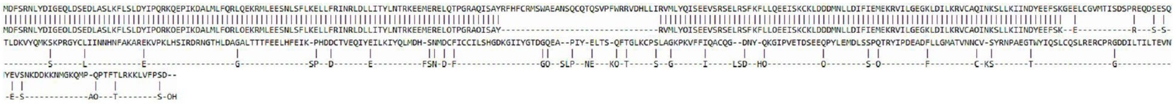

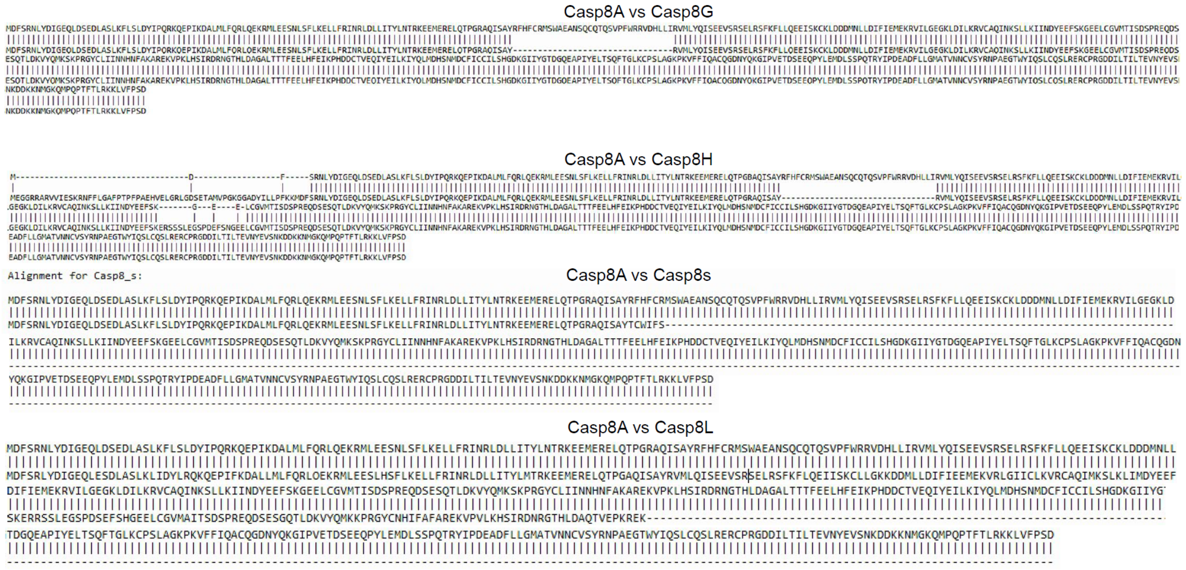

Supplementary Figure S1. Caspase-8 amino acid alignment relative to other caspase-8 isoforms.
